# RETRACTION: A Meta‐Analysis Examining the Impact of Intrawound Treatment on Reducing Deep Surgical Site Infections during Instrumented Spine Surgery

**DOI:** 10.1111/iwj.70527

**Published:** 2025-04-18

## Abstract

**RETRACTION:** ZhouL. and XingS. , “A Meta‐Analysis Examining the Impact of Intrawound Treatment on Reducing Deep Surgical Site Infections during Instrumented Spine Surgery,” International Wound Journal 21, no. 4 (2024): e14554, 10.1111/iwj.14554.38151914 PMC10961050

The above article, published online on 27 December 2023, in Wiley Online Library (http://onlinelibrary.wiley.com/), has been retracted by agreement between the journal Editor in Chief, Professor Keith Harding; and John Wiley & Sons Ltd. Following an investigation by the publisher, all parties have concluded that this article was accepted solely on the basis of a compromised peer review process. In addition, the investigation found significant textual overlap between this article and another article by different authors (Imam et al. 2024 [https://doi.org/10.1111/iwj.14470]). The editors have therefore decided to retract the article. The authors did not respond to our notice regarding the retraction.

**RETRACTION:** L. Zhou and S. Xing , “A Meta‐Analysis Examining the Impact of Intrawound Treatment on Reducing Deep Surgical Site Infections during Instrumented Spine Surgery,” International Wound Journal 21, no. 4 (2024): e14554, 10.1111/iwj.14554.38151914 PMC10961050

The above article, published online on 27 December 2023, in Wiley Online Library (http://onlinelibrary.wiley.com/), has been retracted by agreement between the journal Editor in Chief, Professor Keith Harding; and John Wiley & Sons Ltd. Following an investigation by the publisher, all parties have concluded that this article was accepted solely on the basis of a compromised peer review process. In addition, the investigation found significant textual overlap between this article and another article by different authors (Imam et al. 2024 [https://doi.org/10.1111/iwj.14470]). The editors have therefore decided to retract the article. The authors did not respond to our notice regarding the retraction.

